# Monthly Record of Deaths in the City of Buffalo

**Published:** 1858-03

**Authors:** H. D. Garvin

**Affiliations:** Health Physician


ART. VI.— *Report of Mortality in Buffalo for the month of Jan.*, 1858. By H. D. Garvin, M. D., Health Physician.

| DISEASES. | No. | Males. | Females. | No Sex given. |
|---|---|---|---|---|
| Albuminuria, | 1 | 1 | | |
| Apoplexy, | 2 | 2 | | |
| Asthma, | 1 | 1 | | |
| Bronchitis, | 1 | 1 | | |
| Cephalitis, | 1 | 1 | | |
| Convulsions, | 12 | 5 | 6 | 1 |
| Croup, | 3 | 3 | | |
| Cyanosis, | 1 | | 1 | |
| Diarrhœa, | 1 | | 1 | |
| Enteritis, | 1 | 1 | | |
| Fever, Typhoid, | 2 | 1 | 1 | |
| " Scarlet, | 2 | | 2 | |
| " Puerperal, | 2 | | 2 | |
| Febris Nervosa, | 1 | 1 | | |
| General Dropsy, | 1 | | 1 | |
| Hepatitis, | 2 | 1 | 1 | |
| Hydrocephalus, | 8 | 4 | 3 | 1 |
| Hydrothorax, | 1 | 1 | | |
| Hydrophobia, | 1 | 1 | | |
| Influenza, | 1 | | 1 | |
| Intemperance, | 3 | 1 | 2 | |
| Insanity, | 1 | 1 | | |
| Marasmus, | 2 | 1 | 1 | |
| Metritis, | 2 | | 2 | |
| Old Age, | 5 | | 4 | 1 |
| Phthisis, | 18 | 6 | 8 | 4 |
| Pneumonia, | 9 | 5 | 2 | 2 |
| Premature Birth, | 1 | 1 | | |
| Pharyngitis, | 1 | 1 | | |
| Phlebitis, | 1 | 1 | | |
| Quinzy, | 2 | 2 | | |
| Rubeola, | 3 | 1 | 1 | 1 |
| Still-born, | 7 | 2 | 2 | 3 |
| Schirrhus of Stomach, | 1 | 1 | | |
| Suicide, | 1 | 1 | | |
| Tabes Mesenterica, | 4 | 3 | 1 | |
| Teething, | 5 | 2 | 3 | |
| Uterine Cancer, | 2 | | 2 | |
| Accidental Deaths: | | | | |
| Drowning, | 4 | 3 | 1 | |
| Scalding, | 1 | | 1 | |
| Smothering, | 1 | | 1 | |
| Total, | 144 | 71 | 60 | 13 |

### REGISTER OF MORTALITY—CONTINUED.

### SEXES.

| | |
|---|---|
| Males, | 70 |
| Females, | 61 |
| Sex not given, | 13 |
| Total, | 144 |

### AGES.

| | | | |
|---|---|---|---|
| Still-born, | 7 | Between 20 years and 30 years, | 13 |
| 1 day, | 3 | " 30 " " 40 " | 6 |
| 1 day and 30 days, | 7 | " 40 " " 50 " | 12 |
| Between 1 month and 6 months, | 21 | " 50 " " 60 " | 4 |
| " 6 months and 12 " | 9 | " 60 " " 70 " | 2 |
| " 1 year " 3 years, | 26 | " 70 " " 80 " | 6 |
| " 3 " " 5 " | 5 | " 80 " " 90 " | 1 |
| " 5 " " 10 " | 3 | " 90 " " 100 " | 0 |
| " 10 " " 20 " | 6 | " 100 " | 0 |
| | 87 | | 44 |
| Ages not given, | 13 | | 131 |
| Total, | 144 | | |

### NATIVITIES.

| | | | |
|---|---|---|---|
| American, (white,) | 69 | Prussian, | 1 |
| " (colored,) | 2 | Swiss, | 1 |
| German, | 27 | French, | 1 |
| Irish, | 19 | Scotch, | 0 |
| English, | 4 | Poland, | 0 |
| Canadian, | 0 | Country not given, | 10 |
| Total, | | | 144 |

---

*Poisoning by Twelve Drachms of Laudanum; Recovery.*—Dr. G. C. Gibb records (Lancet, July 25, 1857,) a remarkable case in which twelve drachms of laudanum were swallowed, with a suicidal intent, by a gentleman seventy-two years of age, healthy and temperate, and not in the nebit of using opium. No symptoms of poisoning occurred; the patient passed a restlesss, sleepless night, and nine hours after the laudanum was taken spontaneous vomiting occurred, and under careful treatment he entirely recovered.—*American Journal Med. Science.*

